# WHY LOOKING AT THE FUTURE? FROM PSYCHOLOGICAL TO CONTEXTUAL MOTIVATORS

**DOI:** 10.1093/geroni/igac059.503

**Published:** 2022-12-20

**Authors:** Yaeji Kim-Knauss, Frieder Lang, Christiane Hoppmann

## Abstract

Human beings can represent future events, anticipate future consequences, and act in light of those representations to achieve the most favorable outcomes in the future. Although future-oriented thoughts or behaviors concern distant and delayed rewards than the present and instant ones, their roles in people’s well-being and successful aging have been well-reported. Therefore, what motivates or differentiates such future-oriented thoughts and behaviors has been a central focus in developmental psychology. With a particular interest in future-oriented phenomena regarding age and aging, we look at the roles of psychological or contextual factors that drive views on aging and old-age preparation. Cohn-Schwartz et al. examine how having contacts with older adults benefits self-views on aging via changes in aging stereotypes. Park & Hess explore how importance attached to functioning and perceived control over functioning in different domains predict old-age preparation and compare patterns across different age groups. Fung et al. propose that perceived control, self-relevance, and responsibility for old-age preparation could mediate the well-reported cultural differences in old-age preparation. Rupprecht et al. investigate the adaptivity of approach and avoidance motivation in old-age preparation across different life domains, cultures, and age groups. Kim-Knauss & Lang looks at how the experience of social restrictions during the pandemic functions as a ‘wake-up call’ and thus induces people to engage in old-age preparation. Taken together, we suggest that various psychological appraisals and contexts shape future-oriented thoughts and behaviors, but these may vary across cultures, age groups, and target domains.

